# Multimodal treatment of radiation-associated laryngeal angiosarcoma: A case report and literature review

**DOI:** 10.1097/MD.0000000000046746

**Published:** 2026-01-02

**Authors:** Yiyun Pan, Xiaomei Liu, Xiaoming Nie, Shengkun Wang, Wen Zeng

**Affiliations:** a Department of Oncology, Ganzhou Cancer Hospital, Ganzhou, Jiangxi, China; b Department of Head and Neck Surgery, Ganzhou Cancer Hospital, Ganzhou, Jiangxi, China.

**Keywords:** angiosarcoma, genomic profiling, laryngeal neoplasms, multimodal therapy, radiation-associated neoplasms

## Abstract

**Rationale:**

Angiosarcoma is a rare, highly aggressive malignancy of vascular endothelial origin, marked by rapid local invasion and a high risk of distant spread. Radiation-associated disease is even rarer, and laryngeal involvement is scarcely reported. No standard therapy exists for locally advanced or metastatic cases, and clinical experience is limited. We report a case of radiation-associated laryngeal angiosarcoma and contextualize its diagnosis and management within the literature.

**Patient concerns:**

The patient, a 31-year-old man, was admitted to the hospital, at January 24, 2022, due complaints of generalized bone pain, predominantly in the ribs and lumbar spine, worsening with coughing, along with hoarseness and episodes of choking while drinking, persisting for over 1 month.

**Diagnoses:**

Malignant tumor of the larynx (T3N0M1, stage IVc, AJCC 7th edition); bone metastatic malignancy; splenic metastatic disease; and history of nasopharyngeal carcinoma.

**Interventions:**

Initial treatment with liposomal doxorubicin plus ifosfamide provided partial symptom relief. Genomic testing identified a TP53 mutation, and toripalimab with gemcitabine/nab-paclitaxel produced a partial response lasting 9 months. After progression, sequential targeted agents (anlotinib, lenvatinib, bevacizumab, pazopanib) did not maintain disease control, and the patient died of progressive disease.

**Outcomes:**

The patient died on October 5, 2024, due to respiratory failure, bone marrow suppression, and uncontrolled systemic pain.

**Lessons:**

Radiation-associated laryngeal angiosarcoma is exceptionally rare and portends a poor prognosis. This case illustrates the potential value of genomically informed multimodal therapy integrating chemotherapy, immunotherapy, and targeted agents; in our patient, toripalimab plus gemcitabine/nab-paclitaxel achieved a 9-month partial response. Further studies are needed to establish evidence-based treatment strategies for this understudied disease.

## 1. Introduction

Angiosarcoma is a rare and highly aggressive malignancy arising from the endothelial cells of blood or lymphatic vessels, accounting for approximately 1% to 2% of all soft tissue sarcomas.^[1]^ It most commonly involves the skin, particularly in the head and neck region, whereas laryngeal involvement is exceedingly rare. Angiosarcomas can be classified as either primary or secondary; the latter is often associated with prior exposure to radiotherapy, ultraviolet radiation, or chronic lymphedema.^[2,3]^

Laryngeal angiosarcoma is exceptionally uncommon in clinical practice, with only isolated cases reported in the literature. The disease typically presents insidiously and progresses rapidly, often with local invasion or distant metastasis at the time of diagnosis, leading to significant therapeutic challenges and poor prognosis.^[4]^ While surgery or radiotherapy may be effective in localized disease, there is no established standard of care for advanced or metastatic cases. In recent years, systemic therapies including chemotherapy, targeted therapy, and immunotherapy have been increasingly applied, with some patients achieving temporary disease control.^[5,6]^

In this report, we describe a case of laryngeal angiosarcoma that developed secondary to prior radiotherapy for nasopharyngeal carcinoma. The disease course was aggressive, and multiple treatment strategies were attempted. Through this case, we aim to highlight the diagnostic complexity and therapeutic considerations for this rare malignancy, and to discuss the role of multimodal therapy in its management.

This case adds to the limited literature on radiation-associated laryngeal angiosarcoma by integrating clinicopathologic, imaging, and genomic features and by illustrating a genomics-informed multimodal strategy that achieved a 9-month partial response (PR) after anthracycline/ifosfamide. These hypothesis-generating observations provide practical guidance for treatment sequencing in advanced disease.

## 2. Feature extraction

Contrast-enhanced multidetector computed tomography (CT; United Imaging uCT 760, 128-slice; 1–2 mm reconstructions) was used for lesion measurements on the institutional PACS, and responses were assessed per RECIST 1.1 – PR: ≥30% decrease in the sum of diameters of target lesions; SD: neither meeting PR nor PD; PD: ≥20% increase with an absolute increase ≥5 mm or the appearance of new lesions. Flexible nasopharyngolaryngoscopy was performed with an Olympus CV-170 processor and ENF-VT2 scope. Pathology relied on H&E and IHC on FFPE using an automated stainer (Roche BenchMark XT) with the following antibodies: Vimentin (Fuzhou Maixin kit-0019), CD31 (MAB-0720), CD34 (MAB-1076), ERG (Thermo Fisher MA5-32036), IN1(Abmart Q12824), Ki-67 (MAB-0672), and PD-L1 (VENTANA SP263); the Ki-67 index and PD-L1 tumor proportion score were reported. Tumor genomics used a clinically validated targeted NGS panel (AllNGS™ hybrid capture) on the Illumina NextSeq 500/550 platform; only pathogenic or likely pathogenic variants informed treatment decisions.

## 3. Case presentation

A 31-year-old male first presented in December 2020 to Shanghai Sixth People’s Hospital with a 1-year history of throat discomfort and progressive dysphagia over the past month. He was diagnosed with laryngeal angiosarcoma. The patient received 4 cycles of chemotherapy with doxorubicin (75 mg/m², day 1) and ifosfamide (2.5 g/m², days 1–3), which led to significant improvement in swallowing symptoms. However, he did not adhere to regular follow-up or further treatment thereafter.

On January 24, 2022, the patient was admitted to our hospital with complaints of generalized bone pain, predominantly in the ribs and lumbar spine, worsening with coughing, along with hoarseness and episodes of choking while drinking, persisting for over 1 month. Physical examination revealed normal mouth opening, fair oral hygiene, and a midline uvula. Indirect nasopharyngolaryngoscopy revealed a cauliflower-like mass in the supraglottic region, with right vocal cord fixation and incomplete glottic closure.

Past medical history included nasopharyngeal carcinoma diagnosed in 2010 (cT3N1M0, based on the 2008 staging system), for which he had undergone definitive 3-dimensional conformal radiotherapy (3D-CRT). The radiotherapy protocol included: phase 1 – 36 Gy in 18 fractions to the combined facial and cervical fields; phase 2 – 18 Gy in 9 fractions while sparing the spinal cord and larynx; and phase 3a boost of 14 Gy in 7 fractions to the nasopharynx (x-rays) and positive cervical lymph nodes (electron beam).

**Figure 1.**
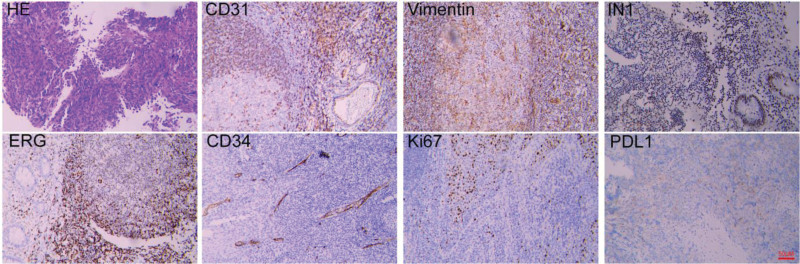
Histopathological and immunohistochemical findings of the laryngeal angiosarcoma. Scale bar = 50 μM.

Histopathological examination revealed a malignant vascular tumor, confirmed by immunohistochemistry showing positive staining for Vimentin, CD31, CD34, ERG, Ki-67 (60%), and PD-L1 (5%), consistent with the diagnosis of angiosarcoma (Fig. 1).

**Figure 2.**
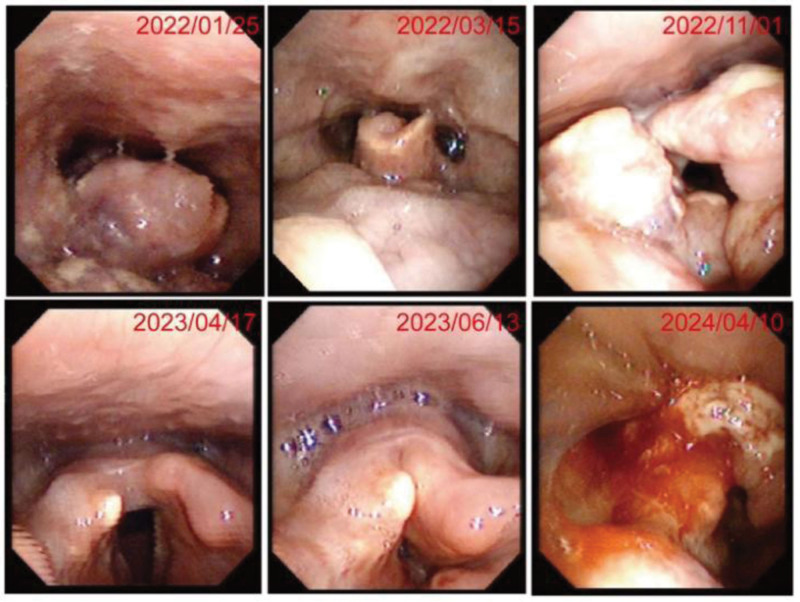
Laryngoscopic findings of laryngeal angiosarcoma during treatment.

**Figure 3.**
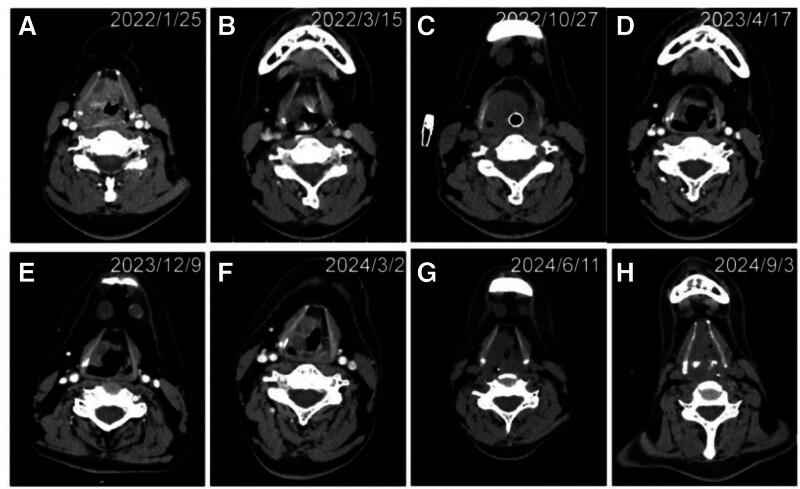
Radiological evolution of laryngeal angiosarcoma during treatment. Time points for each panel: (A) January 25, 2022 (baseline, pre-treatment); (B) March 15, 2022 (early post-initial therapy); (C) October 27, 2022 (mid-treatment follow-up); (D) April 17, 2023 (15-month follow-up); (E) December 9, 2023 (22-month follow-up); (F) March 2, 2024 (25-month follow-up); (G) June 11, 2024 (28-month follow-up); (H) September 3, 2024 (31-month follow-up).

Ancillary examinations included electronic laryngoscopy on January 26, 2022, which demonstrated a supraglottic tumor with features suggestive of malignancy (Fig. 2). Contrast-enhanced CT scan of the oropharynx revealed: a soft tissue mass (15 mm × 32 mm) in the supraglottic region involving the epiglottis, right pyriform sinus, vestibule, right vocal cord, anterior commissure, and right paraglottic space, consistent with a malignant laryngeal tumor; changes in the skull base consistent with prior radiotherapy; multiple intraparenchymal splenic lesions suggestive of metastasis; and abnormal bony lesions in the sternum, thoracolumbar spine, sacrum, and bilateral iliac bones, highly suspicious for bone metastases (Fig. 3).

The admission diagnoses were: malignant tumor of the larynx (T3N0M1, stage IVc, AJCC 7th edition); bone metastatic malignancy; splenic metastatic disease; and history of nasopharyngeal carcinoma.

## 4. Treatment and outcomes

### 4.1. First-line anthracycline/ifosfamide

The patient began systemic treatment on January 27, 2022, receiving 8 cycles of liposomal doxorubicin combined with ifosfamide (administered on 01-27, 02-20, 03-17, 04-11, 05-09, 06-07, 07-14, and 08-31). Serial contrast-enhanced CT scans on March 15, May 7, and July 12 demonstrated reduction in the size of the supraglottic mass to 17 mm × 23 mm, and the disease was assessed as stable disease (SD). On October 27, 2022, the patient experienced acute airway obstruction requiring emergent tracheostomy. CT at that time revealed increased tumor invasion with a mass measuring 18 mm × 28 mm, and the disease was evaluated as progressive disease (PD).

### 4.2. Genomic profiling #1 and immunochemotherapy

The first next-generation sequencing test, performed on November 20, 2022, identified a TP53 exon 4: c.158G > A (p.W53*) mutation, predicting potential sensitivity to gemcitabine and taxane-based therapies (Table 1). Treatment was delayed due to COVID-19 infection. On January 29, 2023, the patient was initiated on a regimen consisting of toripalimab, gemcitabine, and nab-paclitaxel. Follow-up CT on April 17, 2023, showed marked tumor regression to 16 mm × 7 mm, and the response was classified as partial response (PR). However, by October 14, 2023, repeat CT revealed tumor regrowth in the larynx and new pulmonary metastases, consistent with PD.

**Table 1:** Chemotherapeutic agents predicted by the first genetic testing results.

Drug	Sensitivity
Fluorouracil, Tamoxifen, Capecitabine, Gemcitabine, Tegafur-Gimeracil-Oteracil (S-1), Letrozole, Cyclophosphamide, Methotrexate, Paclitaxel, Epirubicin, Anastrozole, other Anthracyclines	High
Irinotecan, Etoposide, Pemetrexed, Docetaxel, Nedaplatin, Raltitrexed, Cisplatin	Moderate
Carboplatin, Oxaliplatin, Vincristine, Vinorelbine	Low

### 4.3. Targeted agents, genomic profiling #2, and pazopanib

Subsequent treatment with targeted therapies including anlotinib, lenvatinib, and bevacizumab was administered. A CT scan on December 8, 2023, indicated further enlargement of metastatic lesions in the liver and spleen, and disease progression was confirmed. A second genetic profiling performed on April 6, 2024, again identified the TP53 c.158G > A mutation and suggested potential sensitivity to pazopanib (Tables 2 and 3). The patient was started on oral pazopanib (800 mg/d) from April 10, 2024. A CT scan on June 11, 2024, showed SD; however, treatment was discontinued due to hematologic toxicity. Bone marrow biopsy confirmed infiltration by tumor cells. Hematologic toxicity was recorded per CTCAE v5.0 and led to treatment discontinuation.

**Table 2:** Chemotherapeutic agents predicted by the second genetic testing.

Drug	Sensitivity
Cisplatin, Carboplatin, Nedaplatin, Lobaplatin, Oxaliplatin, Lobaplatin	High
Methotrexate, Pemetrexed, Raltitrexed, Capecitabine, Fluorouracil, Tegafur, Tegafur-Gimeracil-Oteracil (S-1), Gemcitabine, Cyclophosphamide, Ifosfamide, Docetaxel, Paclitaxel, Albumin-Bound Paclitaxel, Cabazitaxel	
Etoposide, Vinblastine, Vincristine, Vindesine, Vinorelbine, Doxorubicin, Epirubicin, Pirarubicin, Idarubicin, Azathioprine, Mercaptopurine, Thioguanine	Moderate
Anastrozole, Letrozole, Exemestane, Raloxifene, Tamoxifen, Mitomycin	Low

**Table 3:** Targeted agents predicted by the second genetic testing.

Variant	Drug	Tumor type	Therapeutic implication
p.W53*	Buparlisib + Paclitaxel	Head and neck cancer	Potential sensitivity
	Adavosertib	nonspecific cancer type	Potential sensitivity
	Pazopanib + Vorinostat	Sarcoma	Potential sensitivity
	GDC-0575 + Gemcitabine	Sarcoma	Potential sensitivity

### 4.4. Salvage therapy and outcome

**Figure 4.**
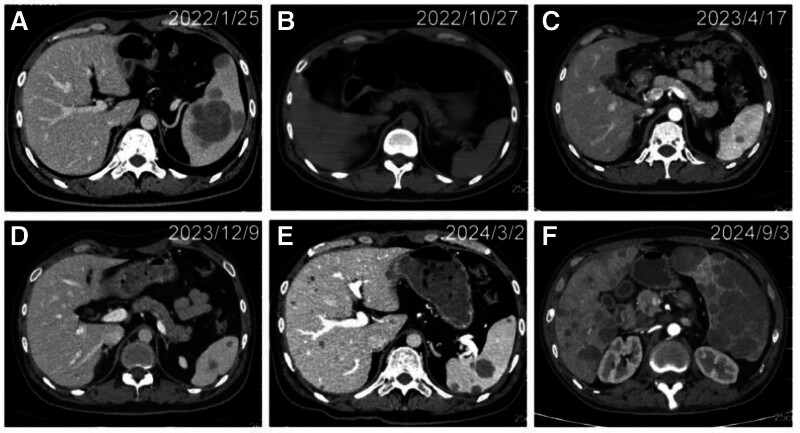
CT evaluation of hepatic and splenic metastases throughout treatment. Time points for each panel: (A ) January 25, 2022 (baseline, pre-treatment); (B) October 27, 2022 (9-month follow-up); (C) April 17, 2023 (15-month follow-up); (D) December 9, 2023 (22-month follow-up); (E) March 2, 2024 (25-month follow-up); (F) September 3, 2024 (31-month follow-up). CT = computed tomography.

**Figure 5.**
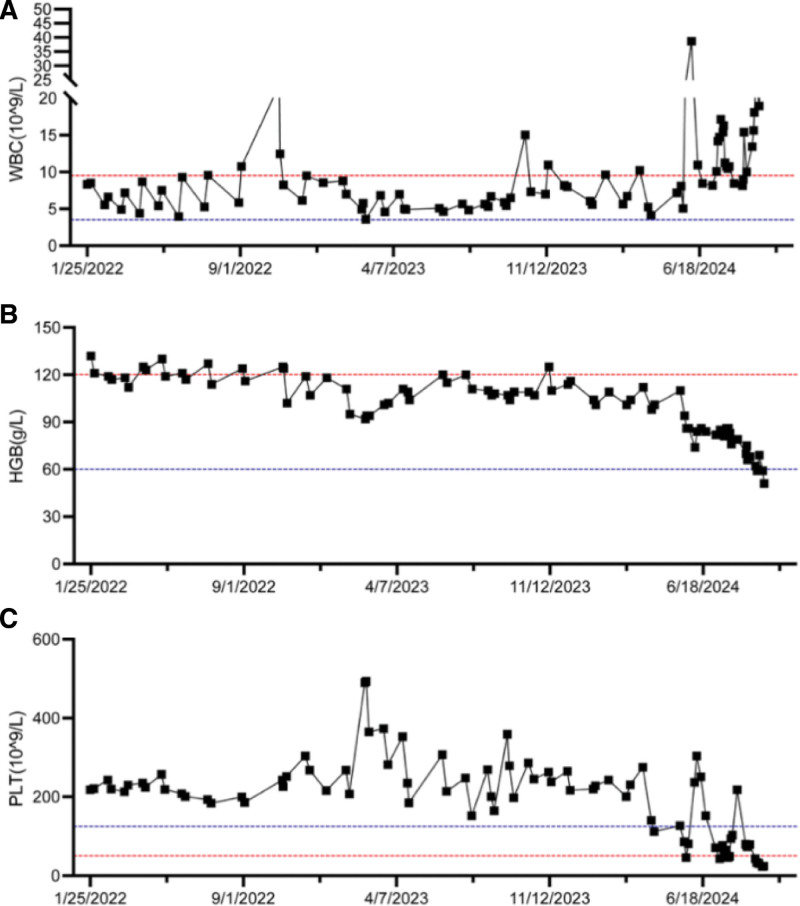
Trends in peripheral blood counts during the course of treatment. Dynamic changes in (A) white blood cell (WBC) count, (B) hemoglobin (Hb), and (C) platelet (PLT) levels are shown throughout the treatment timeline. PLT = platelet, WBC = white blood cell.

On July 24, 2024, the patient was started on weekly paclitaxel, with partial improvement in bone marrow suppression and cancer-related pain. However, follow-up CT on September 3, 2024, revealed a significant increase in the size of the laryngeal mass (46 mm × 37 mm) and widespread progression of metastatic disease. The patient died on October 5, 2024, due to respiratory failure, bone marrow suppression, and uncontrolled systemic pain (Figs. 1–5). Key toxicities included hematologic toxicity during pazopanib therapy resulting in discontinuation. No additional treatment-limiting toxicities were documented in the chart review. Supportive care measures and dose timing are detailed in the therapy timeline (Table 4).

**Table 4:** Systemic therapy timeline and outcomes in the present case.

Period	Regimen	Best overall response	Duration	Reason for discontinuation
January–October 2022	Liposomal doxorubicin + Ifosfamide	SD	−8 mo (imaging SD; airway event October 27, 2022)	PD with airway obstruction
January–October 2023	Toripalimab + Gemcitabine/Nab-paclitaxel	PR	9 mo	PD (laryngeal regrowth and new pulmonary metastases)
November–December 2023	Anlotinib; Lenvatinib; Bevacizumab	PD	<2 mo	PD
April–June 2024	Pazopanib	SD	−2 mo	Hematologic toxicity; BM infiltration
July–October 2024	Weekly paclitaxel	PD	−6 wk	PD → death (October 5, 2024)

PD = progressive disease, PR = partial response.

## 5. Discussion

Angiosarcoma is a rare, highly aggressive malignancy of vascular endothelial origin, accounting for ~1% to 2% of soft-tissue sarcomas.^[1]^ It most often arises in the skin, particularly in the head and neck; laryngeal involvement is extremely uncommon. Angiosarcoma may be primary or secondary. Secondary disease is typically linked to DNA-damaging exposures such as prior radiotherapy, ultraviolet radiation, or chronic lymphedema (Stewart–Treves syndrome).^[2,3,5,6]^ Our patient had a secondary, radiation-associated laryngeal angiosarcoma that developed more than a decade after definitive radiotherapy for nasopharyngeal carcinoma, meeting criteria for radiation-associated sarcoma. Only a handful of laryngeal cases have been reported to date.^[4]^

For localized disease, surgery with adjuvant radiotherapy is standard and improves local control.^[7]^ For locally advanced or metastatic disease, no consensus guideline exists. Most patients receive multimodal systemic therapy – chemotherapy, targeted agents, and immune checkpoint inhibitors – but outcomes remain poor, with a median overall survival of ~5 to 10 months..^[8–10]^

Among systemic therapies, anthracyclines and taxanes are first-line options. Doxorubicin yields an objective response rate (ORR) of ~25%, median progression-free survival (mPFS) 4.9 months, and median overall survival (mOS) 9.9 months.^[11]^ Paclitaxel shows mPFS 4 months and mOS 8 months.^[11]^ In our case, first-line liposomal doxorubicin plus ifosfamide achieved radiologic SD for ~8 months, followed by progression. Initial genomic profiling identified a TP53 c.158G > A (p.W53*) truncation, supporting the use of gemcitabine/taxanes. Toripalimab plus gemcitabine/nab-paclitaxel then produced a PR lasting 9 months. Nab-paclitaxel–based regimens have reported response rates up to 75% with an mPFS of 9.5 months,^[12]^ while gemcitabine monotherapy is generally well tolerated, with an ORR of 54%, mPFS 7 months, and mOS up to 17 months.^[13]^

In recent years, immune checkpoint inhibitors have recently emerged as a promising option for angiosarcoma. In a multicenter phase II study, ipilimumab plus nivolumab achieved an ORR of 25% in metastatic disease, and 60% of responders had cutaneous head-and-neck tumors; 38% of patients maintained PFS ≥6 months.^[14]^ In another cohort of 35 immune checkpoint inhibitor (ICI)-treated patients, the mPFS was 2.7 months and the mOS 9.9 months, with 37% maintaining disease control for ≥16 weeks.^[15]^ Combination ICI therapy, cutaneous subtype, and white ethnicity were associated with better outcomes on multivariable analysis.^[15]^ In a series of 42 previously treated patients, gemcitabine monotherapy yielded an mPFS of 5.4 months and an mOS of 9.9 months.^[16]^ In our case, the toripalimab-based regimen produced a deeper response and longer control than first-line anthracycline-based therapy, underscoring the potential value of genomics-informed multimodal treatment in radiation-associated angiosarcoma.

Multiple targeted agents have been explored. Anlotinib, approved in China as second-line therapy for soft-tissue sarcoma, has shown PFS up to 10 months in a case report.^[17]^ Bevacizumab monotherapy achieved an ORR of 55.8% and a median PFS of 5.5 months.^[18]^ Lenvatinib plus pembrolizumab produced encouraging activity in a single-arm study.^[19]^ In our patient, anlotinib, bevacizumab, and lenvatinib did not achieve disease control. On repeat genomic profiling (TP53 p.W53*), potential sensitivities were suggested to buparlisib + paclitaxel (head and neck cancer), adavosertib, pazopanib + vorinostat (sarcoma), and GDC-0575 + gemcitabine (sarcoma). Considering availability and clinical status, pazopanib – a multi-target tyrosine kinase inhibitor approved for advanced soft-tissue sarcoma – was selected; reported outcomes include mPFS 4.6 months and mOS 12.5 months.^[20]^ n the TAPPAS trial, pazopanib monotherapy yielded a median treatment duration of 4.3 months in angiosarcoma, similar to its performance in soft-tissue sarcoma.^[10]^In our case, pazopanib produced ~2 months of SD before progression.

Several factors may explain the modest durability of immunotherapy and targeted therapy in this case. First, non-cutaneous, radiation-associated angiosarcoma likely lacks the UV mutational signature and high TMB that have been associated with ICI sensitivity in head-and-neck cutaneous disease; PD-L1 expression was low (TPS 5%).^[21]^ Second, radiation-associated angiosarcomas frequently harbor MYC amplification/overexpression, a hallmark of aggressive biology without a direct matched therapy.^[22]^ Third, a VEGF-driven, immunosuppressive tumor microenvironment can blunt antitumor immunity; while antiangiogenic TKIs may transiently normalize vasculature, signaling redundancy often leads to resistance, consistent with the short-lived stability on pazopanib observed here.^[23]^ Finally, advanced disease with bone-marrow infiltration limited dose intensity, further constraining benefit.

In this case, a genomics-informed multimodal regimen – toripalimab with gemcitabine/nab-paclitaxel – achieved a PR lasting 9 months and was clinically tolerable, enabling subsequent sequencing of targeted agents. Responses were assessed per RECIST 1.1. On repeat genomic testing, potential sensitivity to pazopanib was again suggested; pazopanib produced ~2 months of SD before progression with bone-marrow infiltration. Despite salvage weekly paclitaxel, the patient ultimately died from PD with myelosuppression and respiratory failure. Overall, this real-world case adds to the limited evidence in radiation-associated laryngeal angiosarcoma and outlines a pragmatic pathway – early genomic profiling to prioritize a PD-1 inhibitor plus gemcitabine/nab-paclitaxel after anthracycline/ifosfamide, followed by rational sequencing of targeted agents – while remaining exploratory and warranting prospective validation.

## 6. Conclusions

Angiosarcoma is rare and highly aggressive, and no standard therapy exists for advanced disease. In this real-world case of radiation-associated laryngeal angiosarcoma, first-line anthracycline/ifosfamide achieved radiologic SD for ~8 months, while a genomics-informed regimen of toripalimab with gemcitabine/nab-paclitaxel produced a PR lasting 9 months; pazopanib yielded ~2 months of stability. These findings outline a practical sequencing approach – early genomic profiling to prioritize PD-1 blockade plus gemcitabine/nab-paclitaxel after anthracyclines, followed by rational selection of targeted agents. Although exploratory and non-confirmatory, they highlight the value of genomic testing in guiding treatment for this rare entity and define testable hypotheses for future studies.

## Author contributions

**Conceptualization:** Yiyun Pan, Wen Zeng.

**Data curation:** Xiaomei Liu, Xiaoming Nie.

**Funding acquisition:** Yiyun Pan.

**Visualization:** Xiaoming Nie, Shengkun Wang.

**Writing – original draft:** Wen Zeng.

**Writing – review & editing:** Yiyun Pan.
